# The Use of Genus-Specific Amplicon Pyrosequencing to Assess *Phytophthora* Species Diversity Using eDNA from Soil and Water in Northern Spain

**DOI:** 10.1371/journal.pone.0119311

**Published:** 2015-03-16

**Authors:** Santiago Català, Ana Pérez-Sierra, Paloma Abad-Campos

**Affiliations:** 1 Instituto Agroforestal Mediterráneo, Universitat Politècnica de València, Camino de Vera s/n, Valencia, Spain; 2 Forest Research, Alice Holt Lodge, Farnham, Surrey, United Kingdom; Agriculture and Agri-Food Canada, CANADA

## Abstract

*Phytophthora* is one of the most important and aggressive plant pathogenic genera in agriculture and forestry. Early detection and identification of its pathways of infection and spread are of high importance to minimize the threat they pose to natural ecosystems. eDNA was extracted from soil and water from forests and plantations in the north of Spain. *Phytophthora*-specific primers were adapted for use in high-throughput Sequencing (HTS). Primers were tested in a control reaction containing eight *Phytophthora* species and applied to water and soil eDNA samples from northern Spain. Different score coverage threshold values were tested for optimal *Phytophthora* species separation in a custom-curated database and in the control reaction. Clustering at 99% was the optimal criteria to separate most of the *Phytophthora* species. Multiple Molecular Operational Taxonomic Units (MOTUs) corresponding to 36 distinct *Phytophthora* species were amplified in the environmental samples. Pyrosequencing of amplicons from soil samples revealed low *Phytophthora* diversity (13 species) in comparison with the 35 species detected in water samples. Thirteen of the MOTUs detected in rivers and streams showed no close match to sequences in international sequence databases, revealing that eDNA pyrosequencing is a useful strategy to assess *Phytophthora* species diversity in natural ecosystems.

## Introduction

In recent years the increase of global plant trade and human movement have promoted the risk of introduction of invasive plants and exotic pathogens [1–3]. Biological invasions operate globally and are considered to be the second cause of biodiversity loss after direct habitat alteration and destruction. In this context, *Phytophthora* species are of particular importance worldwide as they are major pathogens in agriculture, horticulture and forestry causing important economic and ecological losses.

Environmental DNA (eDNA) from samples such as soil, water, air or permafrost is a complex mixture of genomic DNA from living cells and extracellular DNA from natural cell death from many different organisms [4]. In recent years, the examination of the eDNA in ecological studies has been used to characterize microbial and fungal communities using high-throughput sequencing (HTS) technologies [5–10] and to detect alien species in soil samples [11]. eDNA has also been used successfully to detect low density populations in freshwater environments [12], lakes [13], [14] and aquifers [15]. Most studies have focused on the detection of all organisms present in environmental samples using rDNA genes [16–19] but only a few have targeted only one organism/genera [20], [21].

Traditionally, different methods have been used for the detection of *Phytophthora* species based on isolation on selective media or using different baiting techniques. However, these techniques are time-consuming, and sometimes produce false negatives due to the low inoculum available. Furthermore, identification based on morphological characteristics requires specific taxonomical expertise and a considerable effort. In contrast, different molecular-based methods have been developed and applied for the detection of *Phytophthora* spp. in environmental samples. These methods allow fast and accurate pathogen detection and identification even when the inoculum amount is low, and quantitation of specific *Phytophthora* pathogens is also possible using Real-Time PCR assays [22–25]. A membrane-based oligonucleotide array was developed for the detection of 98 described and 15 undescribed species of *Phytophthora* [26]. Cloning was also applied to assess the *Phytophthora* communities present in soil and water samples [27], but the low throughput of the technique lowers the probability of detecting rare or newly introduced species with low inoculum levels. Different studies have applied HTS for the detection of *Phytophthora* species in soil samples [10], [11], [28], [29], but not, to date, for water.

Increasing economic and environmental losses caused by invasive *Phytophthora* species in natural ecosystems justifies the implementation of an efficient and rapid technique for their detection and accurate identification. In this study, genus-specific primers were adapted to assess *Phytophthora* species diversity in natural ecosystems using high-throughput amplicon pyrosequening of eDNA from soil and water environments, based in the polymorphic and widely accepted barcoding target Internal Transcribed Spacer 1 (ITS1), and validated with a control reaction with DNA of pure cultures. The objective of this study was to apply HTS to investigate the presence of *Phytophthora* in different plant communities in natural forests, plantations and aquatic environments in the north of Spain.

## Material and Methods

### Culture samples

Pure cultures of *Phytophthora gonapodyides* (PS-1512), *P*. taxon PgChlamydo (PS-1510), *P*. *lacustris* (PS-1513), *P*. *cryptogea* (PS-1584), *P*. *citrophthora* (PS-1544), *P*. *plurivora* (PS-1514), *P cambivora* (PS-1556) and *P*. *cinnamomi* (PS-1520) were obtained from the fungal culture collection of the Instituto Agroforestal Mediterráneo, Universitat Politècnica de València (Spain). The identities of these cultures were previously determined by sequencing of ITS region.

### Sampling areas and environmental samples

Two geographical areas were studied. Villanúa (Central Pyrenees, Aragón, Spain) which was selected as a pure European silver fir (*Abies alba*) forest (42°04′13″N, 0°03′13″W) and Irati Forest (42°56′-43°00’N, 1°10′-0°58’E) located in northern Spain (Western Pyrenees, Navarra, Spain). The main vegetation type of Irati Forest is either native beech stands (*Fagus sylvatica*) or mixed beech and European silver fir (*Abies alba*) forest, although it is possible to find recent plantings of Lawson Cypress (*Chamaecyparis lawsoniana*) or Douglas fir (*Pseudotsuga menziesii*) in some areas.

Special authorization for sampling in the protected area of Irati Forest was granted by the Administración Forestal del Gobierno de Navarra. No special permit was required for sampling in Villanúa. This study did not involve endangered or protected species.

### Soil samples

In Villanúa six soils from beneath European silver fir (AS) were collected in June 2012. In Irati Forest, soil samples were collected in October 2012 from 24 different locations selected according to their main vegetation community. The number of samples per vegetation type was proportional to their area in the forest: ten samples were collected from beech stands (F), six from Douglas fir (PS), five from European silver fir (AB), and three from Lawson Cypress (CH). Each sample consisted of soil subsamples from six different points selected at random that were mixed together (approx. 3 kg of soil/sample). Samples were collected around the trees at 1 meter distance from the main trunk by digging to a depth of about 30 cm.

### Water samples

In total 15 water samples were analyzed, two water samples from Villanúa (R1) and 13 water samples from rivers and streams in Irati Forest (R2) during October 2012. Samples were taken after several days of rain. Water samples (10 L) were collected from rivers and streams using plastic containers. The water was filtered *in situ* using a clean knap-sack sprayer to pump the water fitted with a 47 mm polypropylene Swinnex filter holder (Millipore Corp. Bedford, MA, USA) with a 5 μm pore-size autoclaved filter (cellulose acetate and cellulose nitrate mixture) (SMWP04700, Millipore, Ireland). Initially, the collected water was filtered into the sprayer through a 100 μm pore size mesh to remove any debris. Then the water was pumped using three filters per 10 L of water. Each filter was carefully removed with a pair of sterilised tweezers and cut in half with disinfested scissors. The filter was kept in a sterilized 15 ml polypropylene tube (Deltalab S.A., Barcelona, Spain). The tubes were kept in a cool-box during field sampling and in the freezer (-20°C) once in the laboratory. The sprayer was washed with 70% ethanol and rinsed with water thoroughly before sampling in each location.

### DNA extraction

Each soil sample (up to 3 kg) was well mixed and homogenized by sieving (2 mm mesh size), and 50–80 g was lyophilized overnight and then crushed using FRITSCH Variable Speed Rotor Mill-PULVERISETTE 14 (ROSH, Oberstein, Germany). Samples were maintained at 5°C until DNA extraction.

Up to three subsamples were taken from each soil sample, and total genomic DNA was extracted from 300 mg of each subsample using the ZR Soil Microbe DNA MiniPrep (Zymo Research, Irvine, USA) following the manufacturer’s instructions but with the final elutions in 50 μL of elution buffer instead of in a 100 μL of elution buffer.

Filters from water samples (3 filters/sample) were first frozen at −80°C and then disrupted using the TissueLyser LT compact bead mill (Qiagen, UK). DNA was extracted from each of the disrupted filters using the E.Z.N.A. Plant Kit (Omega Bio-tek, Doraville, USA) according to the manufacturer’s recommendations but with the final elutions in 50 μL of elution buffer instead of in a 100 μL of elution buffer.

DNA was extracted from pure cultures using the E.Z.N.A. Plant Kit (Omega Bio-tek, Doraville, USA) according to the manufacturer’s recommendations.

### Control reaction

Each of the genomic DNA from cultures was diluted 10, 100 and 1000 times and amplified separately using a SYBR green real-time PCR assay with the *Phytophthora*-specific primers 18Ph2F and 5.8S-1R [27]. DNAs presenting similar Cycle Threshold values were mixed together to account for differences in ITS copy number.

### Amplicon library generation and 454-pyrosequencing

Amplicon libraries were generated using a nested PCR approach using a Hot Start polymerase (Dominion MBL, Córdoba, Spain). In the first PCR round the *Phytophthora*-specific primers 18Ph2F and 5.8S-1R were used [27]. For the second PCR round, fusion primers were designed following the GS Junior System Guidelines for Amplicon Experimental Design [30], selecting the unidirectional sequencing protocol for library construction (Lib-L chemistry for emulsion PCR, emPCR, ‘One-Way Reads’). The template-specific sequence of the forward fusion primer was the universal ITS6 primer [31] (5'-A-KEY-MID-ITS6–3'), while that of the reverse fusion primer was the same reverse primer used in the first PCR round (5’-B-KEY-5.8S-1R-3’), where A and B represent the pyrosequencing adaptors and the multiplex identifier (MID) was added for postsequencing sample identification. In the case of soil samples, 1 μL of genomic DNA was used in the first PCR round and in the case of water samples the genomic DNA was diluted ten times (approximately 0.2–2 ng was used as template in both DNA sources). The PCR products from the first round were diluted 10 times (soil samples) or 100 times (water samples) for the second PCR round, and in this case, 1 μL was used as a template. The PCR conditions were: 1 cycle of 95°C for 2 min, 30 cycles (1st round) or 25 cycles (2nd round) of 95°C for 20 s, 60°C for 30 s, 72°C for 30 s and a final extension of 72°C for 7 min. For the control reaction, 1 μl of genomic DNA was used in the first PCR round (30 cycles), and 1 μl of PCR product was used as template in the second round (25 cycles).

The PCR products were visualized in a Bioanalyzer 2100 (Agilent Technologies, Palo Alto, CA, USA). The amplicons from soil and pure culture samples were double purified using the Agencourt AMPure XP Bead PCR Purification protocol (Beckman Coulter Genomics, MA, USA). However, in the case of water samples a different method was used with the aim to avoid non-specific products corresponding with other organisms with longer ITS1 fragments than *Phytophthora*, as well as chimeras, which could reduce the quality of the sequencing. Each PCR product from water samples was cleaned using E-Gel SizeSelect (Invitrogen, Burlington, ON, Canada) and the bands between 290 and 450 bp were recovered for sequencing.

After purification, the amplicons were visualized in a Bioanalyzer 2100 and quantified by fluorimetry using Quant-iT PicoGreen kit (Invitrogen Molecular Probes, Eugene, Oregon, USA). The three PCR products from each soil and water samples were mixed together based on their concentration to obtain a single product. The 45 resultant amplicons from the seven areas (each area identified with an individual MID) were pooled at equal concentrations for sequencing.

Amplicon libraries were sequenced in a GS Junior 454 system (Roche 454 Life Sciences, Branford, CT, USA) by the Sequencing and Genotyping Service from the University of Valencia (Burjassot, Spain). Three different emPCR conditions were tested in three different sequencing runs. The first run was performed following Roche’s standard protocol for emPCR amplification for the ‘One-Way Reads’ experimental design, the second run was using the same protocol but removing the larger amplicons by E-Gel SizeSelect, and in the third run, the emulsion was performed following the short length amplicon libraries protocol. Library from pure cultures was sequenced in a fourth sequencing run following the short length protocol.

### Trimming and Molecular Operational Taxonomic Unit (MOTU) clustering

The sequences were first sorted into separate files according to their multiplex identifier (MID) using the sfffile script included in the Roche Newbler package (http://www.454.com/products/analysis-software/). Sequences from each SFF file were extracted using the sff_extract script (http://bioinf.comav.upv.es/sff_extract/index.html) to generate single FASTA, XML and quality files. FASTA and quality files were combined into a single FASTQ file using a Python script (Python version 2.7, Python Software Fdn) and then opened in FastQC software [32] to examine the length and quality of reads.

Reads were trimmed based on quality scores using Lucy software [33]. Sequence length shorter than 100 bp and low quality reads (average read quality below 20) were not considered for analysis. Sequences from the three sequencing runs were combined in a single FASTA file after quality trimming. A new Python script was created to trim the 3’ end (B adaptor, key and reverse primer) and the 5’ end (key, MID and forward primer). This script included a new step to remove sequences shorter than 100 bp after the second trimming.

Different score coverage threshold values were tested for MOTU clustering with a custom-curated database including 146 ITS1 sequences of described and new *Phytophthora* taxa, and the sequences of species used in the control reaction. The resultant FASTA file from each library was clustered with a length coverage threshold of 90% and a score coverage threshold of 99% (-L 0.9 -S 99) using blastclust software [34]. After clustering, a custom Python script was used to convert each MOTU from the output clustering list file into an individual FASTA file, sorting MOTUs in decreasing order of abundance, with a step to remove unique sequences (singletons), and then were aligned by MUSCLE [35]. Each alignment was manually checked in Seaview software [36], and finally, a consensus sequence was generated from each MOTU with the aim to reduce homopolymer and sequencing errors.

The consensus sequences of the MOTUs were identified using the BLAST tool in the GenBank database [34], the *Phytophthora* Database [37] and a custom-curated database comprising Sanger sequences showing a non-mixed chomatogram obtained from environmental samples and culture collections maintained at the Instituto Agroforestal Mediterráneo, Universitat Politècnica de València (Spain). A single sequence was selected at random in those MOTUs that had two reads. Consensus sequences were subjected to phylogenetic analysis to confirm the results obtained from BLAST searches.

## Results

### Sequencing throughput and quality control

The three different emPCR conditions tested produced 1,142 good quality reads in the first run, 14,289 (10,233 used in the present study) in the second and 140,767 reads in the third run.

The total data set from the three sequencing runs comprised 152,142 good quality sequences. The results obtained for each library were: 7,206 sequences for library 1 (R1), 47,419 for library 2 (R2), 13,543 for library 3 (CH), 19,796 for library 4 (PS), 21,982 for library 5 (AB), 26,527 for library 6 (F) and 15,669 for library 7 (AS). After trimming 151,311 sequences were considered for analysis. The average quality of the filtered sequences was of 36 and the average read length was 306 bp.

### Score coverage threshold analysis

A reference database comprising 146 ITS1 sequences of described and new *Phytophthora* taxa was used to test the optimal threshold value. A FASTA file containing the ITS1 sequences was created and clustered at different threshold values. Clustering at 100%, or at 99.5%, separated the higher number of *Phytophthora* species (S1 Fig.). Furthermore, based on ITS1 sequences it was not possible to separate 10% of the species using 100% score coverage threshold.

Using 99% coverage threshold allowed the separation of the highest number of *Phytophthora* species in the control reaction as shown in S2 Fig. Clustering at 99.5 or 100% also separated the eight *Phytophthora* species. However, the number of singletons increased exponentially due to the presence of sequencing and homopolymer errors, producing a high loss of data. Therefore, for all the analysis a 99% of score coverage threshold was used for MOTU clustering.

### Control reaction

A total of 6,698 sequences were generated from the control reaction comprising eight *Phytophthora* species. After quality control, 6,683 sequences were considered for analysis. Applying the 99% score coverage threshold previously calculated a total of 555 MOTUs, including 529 singletons, were obtained. The MOTUs with highest number of sequences corresponded to the eight *Phytophthora* species. The identity of the consensus sequences with Sanger reads was the 100% or 99.5% in all cases. Mismatches were, in all cases, based on homopolymeric regions. The read distribution from each species is shown in Table 1.

**Table 1:** BLAST results of the clustered sequences from the control reaction applying a barcoding threshold value of 99%.

MOTU	Species	Number of Reads	ID with Sanger reads (%)	Error
**1**	***P*. *gonapodyides***	**1190**	**100**	-
**2**	***P*. *cryptogea***	**1025**	**100**	-
**3**	***P*. *cambivora***	**980**	**100**	-
**4**	***P*. taxon *PgChlamydo***	**884**	**100**	-
**5**	***P*. *colocasiae***	**879**	**99.5**	**PolyT**
**6**	***P*. *lacustris***	**604**	**99.5**	**PolyT**
**7**	***P*. *cinnamomi***	**434**	**100**	-
**8**	***P*. *plurivora***	**121**	**99.5**	**PolyT**
9	*P*. *gonapodyides*	3	98	PolyA, -/T, -/G, -/C
10	Chimera	2	-	-
11	*P*. taxon *PgChlamydo*	2	96	A/-, PolyA, T/-, PolyG, T/-, T/-, G/-, -/T, -/T
12	*P*. *gonapodyides*	2	98	PolyA, -/G, C/T, -/G
13	Chimera	2	-	-
14	*P*. *gonapodyides*	2	98	G/A, T/-, G/-, T/-, G/-
15	*P*. *cambivora*	2	97	C/T, T/C, T/A, T/A, C/T, T/A
16	*P*. *gonapodyides*	2	98	PolyA, A/-, PolyT, PolyA
17	*P*. *gonapodyides*	2	97	PolyG, T/C, C/T, T/-, T/-, G/A
18	*P*. *gonapodyides*	2	98	C/T, A/G, T/C, T/C
19	*P*. taxon *PgChlamydo*	2	97	A/-, -/A, T/C x3
20	*P*. *colocasiae*	2	98	PolyA, G/-, PolyT x2
21	*P*. *cryptogea*	2	99	G/A, G/T
22	*P*. *colocasiae*	2	98	PolyA, G/-, T/-
23	*P*. *colocasiae*	2	96	A/-, PolyA, T/A, A/C,-/T, PolyT
24	*P*. *plurivora*	2	98	PolyT, T/C, A/T
25	*P*. *plurivora*	2	97	PolyA, PolyT, T/C, A/T
26	*P*. *plurivora*	2	98	PolyT x3

Results only include MOTUs with more than two sequences. Identity and mismatches with Sanger reads are also indicated. MOTUs with higher number of sequences are in bold.

### Analysis of MOTUs from soil eDNA samples

**Fig 1:**
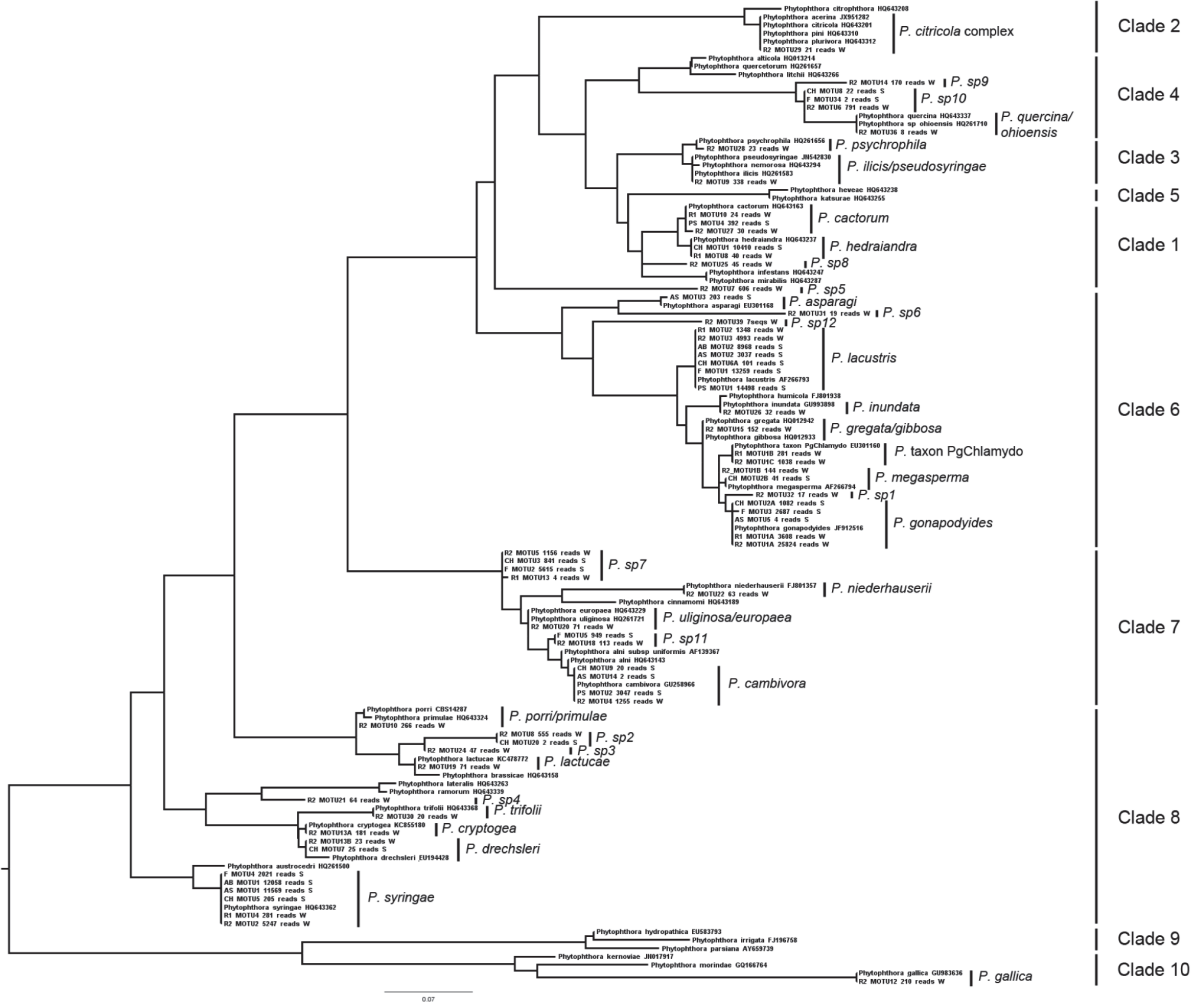
Unrooted phylogram based on nuclear ITS1 rDNA sequence analysis constructed with maximum likelihood approach. Each of the MOTU includes library precedence (R1, R2, CH, AB, F, PS or AS), number of MOTU resulted in the clustering at 99% of each library, number of reads and source (W, water; S, soil). Vertical bars indicate the *Phytophthora* species. MOTUs corresponding with undescribed species are indicated as “sp” (from sp1-sp12).

A total of 96,895 reads which passed quality control from soil samples (sampling areas CH, PS, AB, F and AS) were considered for analyses. Clustering of 96,895 reads resulted in 8,151 MOTUs, including 8,114 singletons, which were discarded for the analysis. The 37 nonsingleton MOTUs corresponded to 13 *Phytophthora* species, included in clades 1, 4, 6, 7 and 8 (Fig. 1) from four plant communities: *A*. *alba*, *C*. *lawsoniana*, *P*. *menziesii* and *F*. *sylvatica*.

The specificity of the technique for the detection of *Phytophthora* in soils was 98.46%. The non-*Phytophthora* MOTUs (1.54% of nonsingleton clustered reads) matched with other plant pathogen genera such as *Pythium* and *Hyaloperonospora* (Table 2).

**Table 2:** Read distribution of species other from *Phytophthora* based on the ITS1 after BLASTn of the consensus sequences performed against GenBank. Reads from MOTUs with more than 99% similarity are in bold.

	Villanua		Irati Forest					
Putative species detected non- *Phytophthora*	R1	AS	R2	CH	F	AB	PS	Total reads per species
*Hyaloperonospora parasitica*	**361**							361
*Hyaloperonospora sp*. *1*							894	894
*Hyaloperonospora sp*. *2*	248							248
*Hyaloperonospora sp*. *3*			62					62
*Hyaloperonospora sp*. *4*			117					117
*Hyaloperonospora sp*. *5*	161							161
*Hyaloperonospora sp*. *6*					198			198
*Bremia lactucae*			**5**					5
*Peronospora aparines*	**165**							165
*Peronospora aestivalis*			**232**					232
*Peronospora glomerata*			**150**					150
*Pythium sp 1*	7		2		6			15
*Pythium sp 2*		21						21
*Pythiaceae 1*	40							40
*Pythiaceae 2*	10							10
*Pythium minus*	**2**			**250**				252
**Total reads per sampling area**	994	21	568	250	204	0	894	

### Analysis of MOTUs from water eDNA samples

A total of 54,416 reads from water eDNA from the sampling areas R1 and R2 which passed quality control were considered for analyses. Clustering of 54,416 reads resulted in 3,646 MOTUs, including 3,548 singletons, which were discarded for the analysis. The 98 nonsingleton MOTUs corresponded to 35 *Phytophthora* species (Fig. 1) from clades 1, 2, 3, 4, 6, 7, 8 and 10 (Fig. 1). No *Phytophthora* species were detected from clades 5 and 9. The clades with the highest number of species were clade 6 and 8 with nine species in each. Only one species from clade 2 was found, matching with a *Phytophthora citricola*-complex species. The three most abundant MOTUs in water samples were *P*. *gonapodyides* (29,432 reads), followed by *P*. *lacustris* (6,341 reads) and *P*. *syringae* (5,528 reads). Fifteen sequences were obtained of a *Phytophthora* species (named as *Phytophthora* sp. 13) which sequences did not match any sequences in public databases and did not cluster in any of the ITS clades described. The closest match in GenBank was *Phytophthora* sp. REB326-69 (accession no. JX122744) with a sequence homology of 89%. This MOTU was not included in Fig. 1 as it was too divergent.

The specificity of the water eDNA pyrosequencing assay for *Phytophthora* detection was 96.92%. The non-*Phytophthora* MOTUs (3.08% of nonsingleton clustered reads) matched with other plant pathogen genera such as *Hyaloperonospora*, *Bremia*, *Peronospora*, *Pythium* or members of an undetermined Oomycete genus not represented in GenBank (Table 2).

## Discussion

The detection of *Phytophthora* from environmental samples using high-throughput amplicon pyrosequening of eDNA has shown high specificity and can be used hereafter to assess *Phytophthora* diversity in natural ecosystems.

Clustering at 100% was the best criteria to separate most of the species in the reference database. Other studies [38] adopted a barcoding threshold of 98% to assign most reads. However, in the current study we included a control species mixture and a 99% threshold was required to discriminate between closely related species resulting in the separation of 20% more species.

For the control reaction data, clustering at 99% of similarity was the minimum value able to separate all of the species with the minimum number of reads lost. Clustering at 99.5% or 100% resulted in the loss of reads due to the presence of homopolymer and sequencing errors that were discarded as singletons. Values below 99% were insufficient to separate *P*. *gonapodyides* and *P*. taxon PgChlamydo in separate MOTUs. In this study, clustering at 99% similarity allowed the separation of most species from clade 6 found in environmental water samples and reduced the risk of false MOTUs produced by sequencing errors. Although the pooling for the control reaction was made by mixing the DNAs based on similar CT values, the number of sequences obtained of *P*. *plurivora* was lower than expected and this could be a potential issue to consider when interpreting the results.

The limitations of the ITS1 region for *Phytophthora* taxonomic identification are particularly evident in clade 1, in clade 2 with the *P*. *citricola* complex [39] and in clade 6 which includes many aquatic species where many species are identical or differ by 1 bp in ITS1. Applying a similarity of 100% between sequences, it is still not possible to separate 10% of the species in the reference database, including species from clade 1 (*P*. *infestans*, *P*. *ipomoeae*, *P*. *mirabilis*, *P*. *andina*), from clade 2 (*P*. *capensis*, *P*. taxon emanzi) or clade 6 (*P*. *gibbosa*, *P*. *gregata*). Despite these limitations it remains a powerful tool for discrimination of most known species as well as the identification of new ones.

The conditions for emPCR amplification (2.2 million copies of template and 80 μL of Amp Primer A) used in the first and second sequencing runs resulted in light scattering into nearby wells and caused their elimination due to signal processing filtering. The third sequencing run was performed following guidelines for Short Length Libraries which reduced the amount of Amp Primer A in the Live Amp Mix from 80 to 20 μL. Although environmental ITS1 amplicons length did not match the Short Length Libraries definition (amplicons of 340 bp instead of amplicons <250 bp), the use of this protocol increased 100 fold the throughput in comparison with the first pyrosequencing run.

Similar studies based on pyrosequencing in soil samples from beech forests in France revealed the presence of only four *Pythiaceae* species (corresponding to the 0.8% of the sequences from the dataset), represented by two *Phytophthora* species: *P*. *plurivora* and an unidentified *Phytophthora* species from clade 7a [10]. Their methodology for library generation was based on a nested PCR approach, where the first PCR was based on the amplification of the whole ITS region with the primer pair ITS6/ITS4 [31], [40] and pyrosequencing was based on the ITS6/ITS7primers [31]. This pyrosequencing strategy revealed lower efficacy and specificity for *Phytophthora* detection in comparison with the methodology implemented in this study. The throughput of amplification of large targets, like whole ITS region in genus *Phytophthora* (up to 840 bp), is usually a problem in environmental samples, especially in soils, with abundant humic acids inhibitors and high DNA degradation. The importance of the length of the targeted region is that short DNA fragments (less than 300–400 bp) are usually very slowly degraded which allows their detection in environmental samples [41]. The implementation of large-scale eDNA-based ecological studies is highly dependent on the availability of suitable short metabarcodes [4]. Therefore, using in the first PCR the *Phytophthora*-specific primers as implemented, which include the ITS1 and a small portion of the 18S (amplicon length from 419 to 484 bp), rather than the whole ITS region provides better amplification results for metagenomics studies.

In contrast, other researchers [11] applied the same ITS6/ITS7 primers for pyrosequencing without using a nested approach, revealing the presence of 15 *Phytophthora* species in soils from chestnut forests in Italy. Their assay was very specific for *Phytophthora* species detection, where 78.8% of sequences (9,167 of 11,637 reads) matched *Phytophthora*.

In this study, a nested approach was used in order to yield suitable PCR products since no amplification was obtained with a single round PCR [27]. Nested PCR increases sensitivity and specificity compared to non-nested PCR techniques, but also increases the risk of cross-contamination events. To further reduce the occurrence of false-positives based on cross-contamination extremely care should be taken during DNA extraction, preparation of PCR reactions and post-PCR steps.

From a methodological point of view, soil pre-processing procedures (drying, sieving, lyophilizing, crushing and weighting) are time-consuming in comparison with the water filter processing. Working with water samples allows the processing and amplicon library generation in one day. Pyrosequencing of soil samples revealed lower *Phytophthora* diversity than from water samples, where only thirteen species were detected in five different ecosystems comprising 30 sampling sites. The highest number of species was detected in three sampling sites of *Chamaecyparis* plantations where 10 of the 13 *Phytophthora* species were detected. It is especially surprising that so few species were detected in *Abies alba* soil samples from Irati Forest, where only two species, *P*. *lacustris* and *P*. *syringae* were detected. Similarly within *Pseudotsuga menziesii* associated soil samples where only three species were detected. Pyrosequencing of water eDNA from rivers and streams revealed a hidden and unexpectedly high *Phytophthora* species diversity. A total of 35 *Phytophthora* species were detected, 13 of which could represent potentially novel species. *Phytophthora* species diversity from water samples in Irati Forest comprised all species detected in soil samples from the same sampling area. However, the species detected in water and soil samples in Villanúa were different. This could be due to the fact that a low number of samples were taken from this forest and also due to the different sampling times.

The DNA recovered from the filters is probably composed of DNA originated from living cells (mainly zoospores) and extracellular DNA originated from natural cell death in water. These could be present in the water itself or they could be have originated from washing off the soil after rain periods. Other studies [42] have demonstrated that DNA fragments in freshwater ecosystems persist for less than one month. This indicates that by sampling immediately after a rain event is possible to recover the maximum amount of DNA containing the potential *Phytophthora* community from the forests and water environments. This hypothesis is congruent with the presence of several *Phytophthora* species from most clades in water eDNA, including many plant pathogenic species usually isolated from roots such as *P*. *cambivora*, *P*. *niederhauserii*, *P*. *plurivora*, *P*. *pseudosyringae* or *P*. *syringae*.

Most of the *Phytophthora* species isolated from irrigation reservoirs and natural waterways belong to clade 6. These species, commonly called water moulds, show a strong association with both forest and riparian ecosystems, [43–46] and although some of them can be aggressive tree pathogens like *P*. *inundata* in Spain [47], it is hypothesized that they have a prevalently aquatic and saprophytic lifestyle [48]. In this study 73.6% of the reads belonged to clade 6, which may represent the resident community in rivers and streams. The most common species detected were *Phytophthora gonapodyides*, *P*. *megasperma*, *P*. taxon PgChlamydo, *P*. *inundata* and *P*. *lacustris*, which are commonly isolated by baiting from streams around the world [45], [49–53].

The *Phytophtora*-specific primers [27] used in the current study showed high specificity. In the original study the amplicons were cloned and a total of 260 amplicons were obtained with a complete absence of *Pythium s*. *lato* species. Only a minor cross reaction with two downy mildew phylotypes was found. However, in this study using the same primers with an HTS approach a total number of 152,142 reads were obtained with 97.9% specificity to *Phytophthora*. Only a small percentage (2.1%) of non-*Phytophthora* amplification was obtained, including the genus *Hyaloperonospora*, *Bremia*, *Peronospora* and *Pythium*.

Previous techniques (baiting of environmental samples and cloning) are time consuming and costly, and often are likely to generate false negatives due to the low frequency of some *Phytophthora* species and the inability of unknown Phytophthoras to grow on some artificial media. The technique implemented here overcomes these problems and can produce results in less than one week compared to several weeks or months by conventional techniques which would allow an early response to the threats posed by these pathogens. Although similar techniques have been used in previous studies it is the first time that genus-specific amplicon pyrosequencing has been used to target only *Phytophthora* in soil and water samples, unlocking previously hidden *Phytophthora* communities.

### Accession numbers

The three pyrosequencing runs were combined into a single file and deposited in GenBank-SRA under the accession number SRP027499. Consensus nucleotide sequences of each *Phytophthora* MOTU were deposited in GenBank (accession no. AF132232 to AF132286).

## Supporting Information

S1 Fig Number of MOTUs generated using different score coverage threshold values based on ITS1 using a reference database of *Phytophthora* species. (TIF)

S2 Fig Number of *Phytophthora* species detected and singletons produced per score coverage threshold in the control reaction. (TIF)
