# Supplementary figures and images for: The Use of Genus-Specific Amplicon Pyrosequencing to Assess Phytophthora Species Diversity Using eDNA from Soil and Water in Northern Spain
Source: PLoS One. 2015 Mar 16;10(3):e0119311. doi: 10.1371/journal.pone.0119311 (PMC4361056; doi:10.1371/journal.pone.0119311)

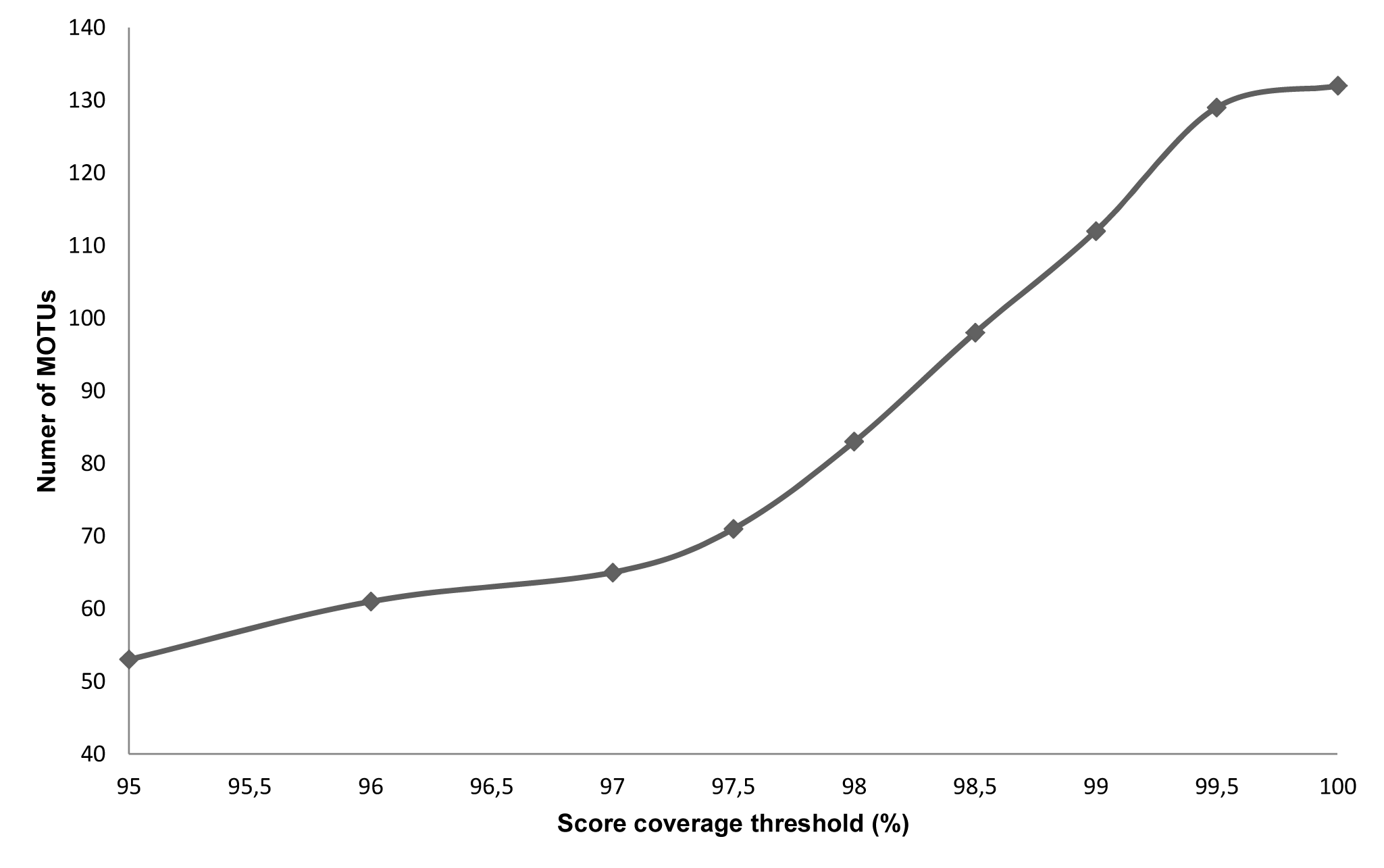

Supplement: S1 Fig — (TIF) [file pone.0119311.s001.tif]

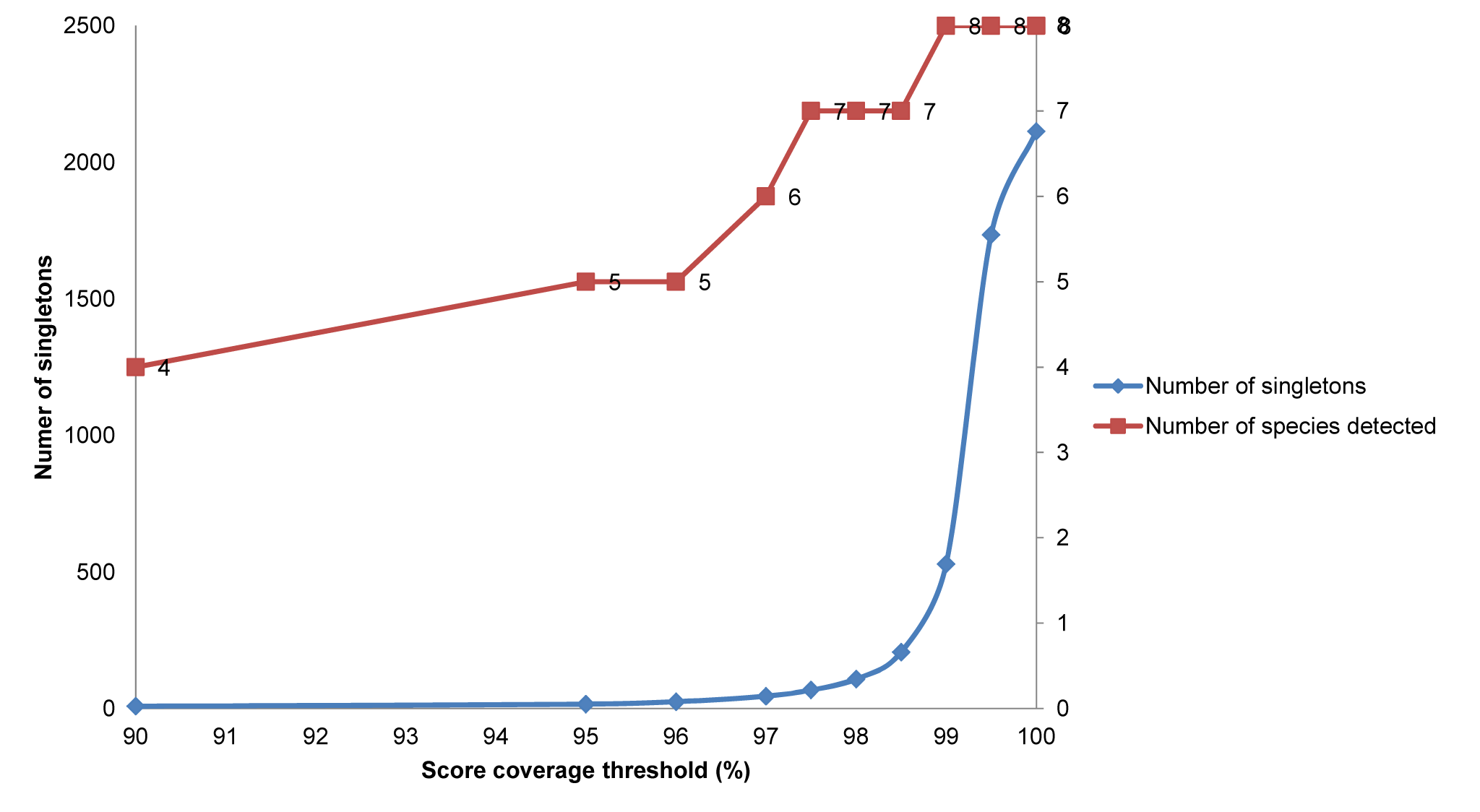

Supplement: S2 Fig — (TIF) [file pone.0119311.s002.tif]
